# *Mycolicibacterium nivoides* sp. nov isolated from a peat bog

**DOI:** 10.1099/ijsem.0.004438

**Published:** 2021-03-01

**Authors:** John L. Dahl, Wayne Gatlin III, Phuong M. Tran, Cody S. Sheik

**Affiliations:** ^1^​ Department of Biology, University of Minnesota Duluth, Duluth, MN 55812, USA; ^2^​ Large Lakes Observatory, University of Minnesota Duluth, Duluth, MN 55812, USA

**Keywords:** *Mycolicibacterium nivoides*, *Mycolicibacterium*, sphagnum peat bog

## Abstract

A fast-growing, non-chromogenic, acid-fast-staining bacterium (DL90^T^) was isolated from a peat bog in northern Minnesota. On the basis of 16S rRNA gene sequence similarity (99.8 % identity with *Mycolicibacterium septicum* and 98 % with *Mycolicibacterium peregrinum*) and chemotaxonomic data (fatty acid content), strain DL90^T^ represents a member of the genus *Mycolicibacterium*. Physiological tests (growth curves, biofilm formation, antibiotic sensitivity, colony morphologies and heat tolerance) and biochemical analysis (arylsulfatase activity and fatty acid profiles) distinguish DL90^T^ from its closest relative *M. septicum*. Phylogenomic reconstruction of the ‘*Fortuitium–Vaccae*’ clade, digital DNA–DNA hybridization (DDH) values of 61 %, and average nucleotide identity (ANI) values of approximately 95 % indicate that DL90^T^ is likely to be diverged from *M. septicum*. Thus, we propose that DL90^T^ represents a novel species, given the name *Mycolicibacterium nivoides* with the type strain being isolate DL90^T^ (=JCM 32796^T^=NCCB 100660^T^).

## Abbreviation

FAME, fatty acid methyl esters.

## 

The genus *Mycobacterium* contains over 188 species and has recently been subdivided into four novel genera, including the fast-growing ‘*Fortuitium–Vaccae*’ clade for which species are designated with the new genus name *Mycolicibacterium* [1]. While Mycobacteria are generally associated with human disease, Mycolicibacteria are typically found in diverse, non-host-associated environments. Previous work has shown that peat bog environments, which are typified by their high organic matter, low pH and anoxic conditions, are particularly rich in mycobacterial species [2]. Given the potential for discovery of new Mycobacteria in these ecosystems, we sampled the Big Bog in northern Minnesota for acid-fast staining cells in sphagnum peat moss, as previously described [3]. Several acid-fast isolates from a pool of 720 mycobacterial candidates were further tested [4]. Candidates for being novel species were paired down by first performing restriction enzyme analysis of PCR products for the genes *rpoB*, *dnaJ* and *hsp65*. Isolates with unique banding patterns were pooled and their PCR products sequenced for reconstruction of concatenated sequences (*rpoB–dnaJ–hsp65*) to produce phylogenetic trees for comparison with known species. From this initial screen a potential novel species was chosen for further characterization and is referred to here as strain DL90^T^.

Isolate DL90^T^ was grown using tryptic soy broth (TSB) or agar (TSA), Middlebrook 7H10 agar supplemented with oleic acid, bovine albumin, sodium chloride, dextrose and catalase (OADC), or Middlebrook 7H9 broth supplemented with albumin dextrose and catalase (ADC). Tween 80 was added to both TSB and 7H9 liquid cultures during growth curve experiments to compare with other characterized mycobacterial isolates obtained from the American Tissue Culture Collection. No Tween 80 was added for experiments testing biofilm formation. fatty acid methyl ester (FAME) analysis was performed by Microbial ID Inc (http://microbialid.com) using DL90^T^ cells grown on TSA, as previously described [5].

**Fig. 1.**
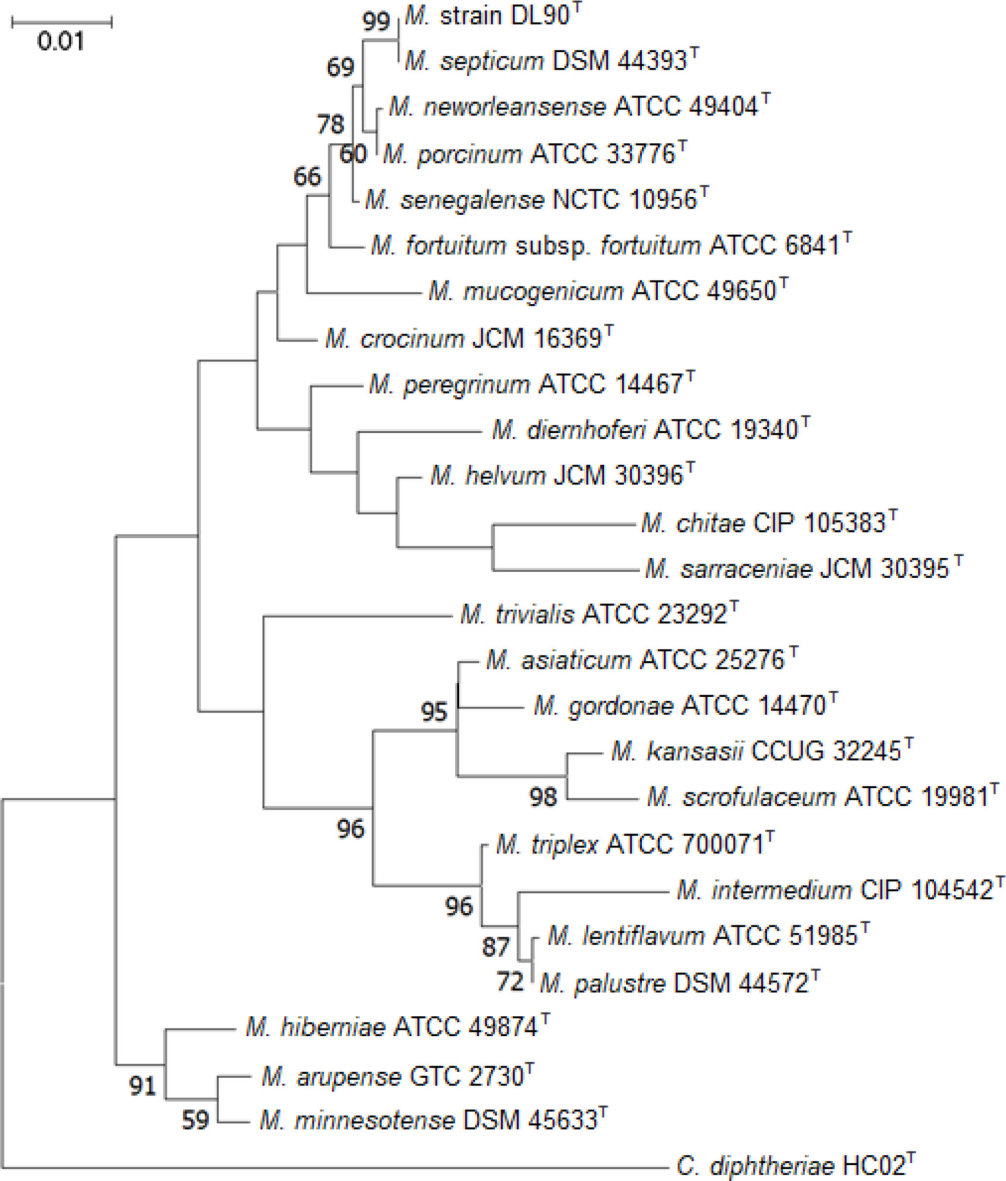
16S rRNA gene maximum likelihood tree indicating the phylogenetic position of isolate DL90^T^ and the most similar mycobacteria species as determined by blast sequence analysis. *Corynebacterium diptheriae* HC02^T^ was used as the outgroup. Units for scale bar are nucleotide substitions per position. Genbank accession numbers for 16S rRNA sequences are listed in Table S1 (available in the online version of this article).

To sequence the DL90^T^ genome, DNA was isolated from liquid-grown cultures (7H9 supplemented with ADC) using NucleoSpin Tissue Extraction kits (Macherey–Nagel). The DNA sequencing library was prepared using a Nextera XT kit (Illumina) and sequenced on the MiSeq platform with 2×300bp v3. Assembly of the genome was done using the Galaxy web platform. Prior to assembly, reads were trimmed using Trimmomatic [6] and quality-checked with FastQC (https://www.bioinformatics.babraham.ac.uk/projects/fastqc/). A *de novo* assembly was performed using Unicycler [7] which uses SPAdes to initially assemble the short reads together [8]. The final assembled genome was deposited with the National Centre for Biotechnology Information (NCBI), with the accession number CP034072.1. Using a recovered 16S rRNA gene from the DL90^T^ genome, we compared DL90^T^ to previously characterized mycobacteria, as previously described [3]. Using near-full length 16S rRNA genes, we created a multiple sequence alignment with clustalx and a maximum likelihood phylogenetic tree was reconstructed with mega7 [9]. Evolutionary history was inferred by the maximum-likelihood method and distances were computed using the Kimura two-parameter distance correction model with a total of 1000 bootstrap replications. Initial tree(s) for the heuristic search were obtained automatically by applying Neighbor-Join and BioNJ algorithms to a matrix of pairwise distances estimated using the maximum composite likelihood (MCL) approach, and then selecting the topology with a superior log likelihood value. The tree with the highest log likelihood (−5693.38) is shown (Fig. 1). Our phylogenetic reconstruction indicates that isolate DL90^T^ is closely related to the species *Mycolicibacterium septicum*, which was also determined by blast sequence analysis [10].

**Fig. 2.**
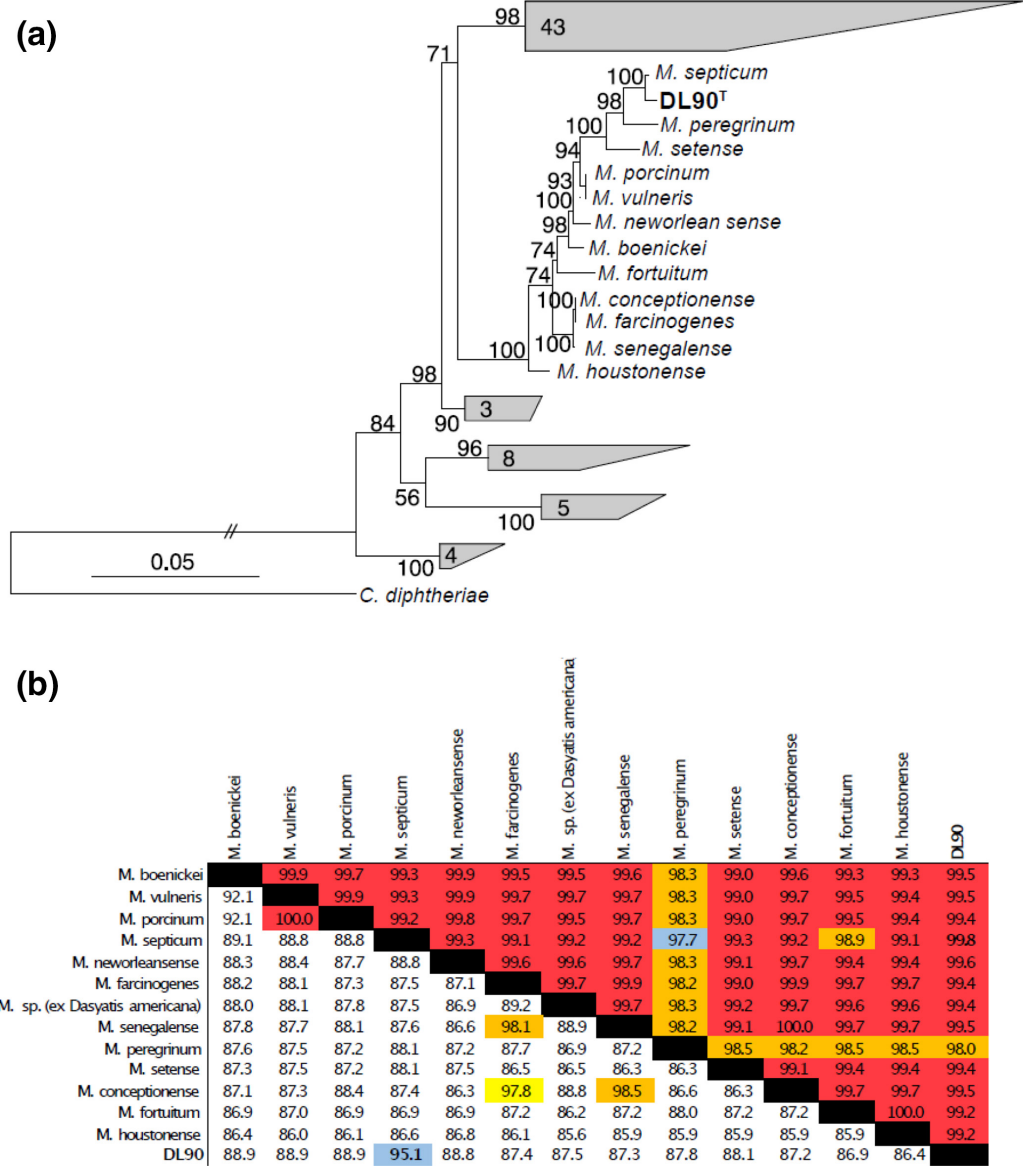
Phylogenomic tree of the ‘*Fortuitum–Vaccae*’ clade of *Mycobacteria* (**a**) and the similarity of 16S rRNA genes (top portion of triangular matrix) and average nucleotide identity (ANI) of genomes (bottom portion of triangular matrix) Units of scale bar are amino acid substituions per position.(**b**). Numbers within the collapsed clades of the phylogenomic tree represent the number of genomes that are within that clade. Phylogenomic reconstruction was based on 15 single-copy genes and the tree was bootstrapped 1000 times with IQ-Tree (see text for full methods). Red highlighted cells represent similarity values >99 %, orange cells 98–99 % similarity, yellow 97–98 %, and blue cells 95–97 % similarity.

To confirm phylogenetic placement of the DL90^T^ genome (Fig. 1), we used a phylogenomic approach to compare the genomes from the ‘*Fortuitum–Vaccae*’ clade of Mycobacteria [1]. Genomes (76 total) from NCBI’s Genbank and Refseq were downloaded and processed using the GToTree pipeline [11]. The pipeline uses Prodigal [12] to call genes and translate to amino acids, HMMER3 [13] to search for marker proteins with HMM models, muscle [14] to create alignments of protein sequences, TrimAl [15] for trimming protein sequence alignments before concatenation, and IQ-Tree [16] for generating a bootstrapped phylogenetic tree. IQ-Tree was run using default settings. We used the Universal_Hug_et_al HMM set which is comprised of fifteen universal single copy genes (Ribosomal_L14, Ribosomal_L16, Ribosomal_L18, Ribosomal_L22, ribosomal_L24, Ribosomal_L2, Ribosomal_L3, Ribosomal_L4, Ribosomal_L5, Ribosomal_L6, Ribosomal_S10, Ribosomal_S17, Ribosomal_S19, Ribosomal_S3_C and Ribosomal_S8), that have been shown to create a robust phylogenetic reconstruction [17]. For the ‘*Fortuitum–Vaccae*’ clade, we found that none of the genome contained the large subunit ribosomal protein (L18), hence this was excluded from the alignment. Phylogenetic placement using the concatenated protein tree indicates, as expected, that the DL90^T^ genome is highly similar to the *M. septicum* DSM 44393^T^ genome and belongs to a larger clade with *M. setense*, *M. neworleansense*, *M. fortuitum* and *M. peregrinum* (Fig. 2a). We found that the phylogenomic placement was congruent with the 16S rRNA gene tree (Fig. 1) and indicated that several of the genomes were highly similar to one another and warranted further investigation.

Comparison of the 16S rRNA gene sequences of DL90^T^ to those of other closely related isolates (Fig. 2b) indicated that all the genomes were highly similar to each other with respect to 16S rRNA genes and would all fall within the same species if a 97 % similarity cutoff were used (Fig. 2b, upper triangle matrix). The high degree of similarity amongst all of the isolates in this clade indicates that the 16S rRNA gene is not a robust measure of species divergence in this clade. Thus, we choose to apply a more robust measure of species divergence, average nucleotide identity (ANI) [18], to measure how similar genomes were to each other. To quantify ANI we used the programme fastANI [19]. Despite the high similarity of the 16S rRNA gene in this clade, the majority of genomes fell below the currently recognized species cutoff threshold of approximately 95 % similarity. The DL90^T^ and *M. septicum* DSM 44393^T^ genomes, which clustered together in the phylogenomic tree, were 95.1 % similar to each other by ANI, indicating that these two genomes are potentially the same species. Initial testing using the online ANI calculator (http://enve-omics.ce.gatech.edu/ani/index), which has a different implementation of ANI than fastANI, indicated that the two genomes were 94.97 % similar [18]. Interestingly, we did identify two instances where genomes were 97–100 % similar to other genomes by ANI, nearly identical in the phylogenomic tree, and highly similar by 16S rRNA genes (Fig. 2a, b, see genomes for *M. porcinum, M. vulneris, M. conceptionense, M. farcinogenes* and *M. senegalense*). This would indicate that either these isolates have a high degree of phenotypic divergence or that they should be reclassified as strains of a single species.

Further genomic comparisons of DL90^T^ to *M. septicum* DSM 44393^T^ revealed that their genomes are similar in size (6 905 961 bp and 6 872 299 bp, respectively) with the DL90^T^ genome slightly larger and containing approximately 80 more genes (Table S2). Although their genome sizes are similar, and DL90^T^ and *M. septicum* DSM 44393^T^ share 5234 orthologous genes, this homology constitutes only 81 % of their genomes. This indicates their genomes have gained and/or lost genes as they have diverged. For comparison, the genome content of DL90^T^ (AAI, orthologous genes and orthologous fraction) varies across the larger clade of closely related genomes (Table S2) and indicates that the life history of these organisms is complicated. Amino acid identity (AAI) values were slightly higher than ANI values at 95.93 similarity (Table S2), however, there are currently no recommended or accepted AAI values to delineate novel species [20]. Digital DNA–DNA hybridization (DDH) produced a reassociation value, using formula two, of 61 % between DL90^T^ and *M. septicum* DSM 44393^T^ which is below the <70 % threshold of hybridization between members of the same species [19]. Comparisons of DL90^T^ with other isolates show very low DDH values (<40 %), which is consistent with our other analyses (Table S2). Nonetheless, these indicate that DL90^T^ is potentially sufficiently diverged at the genomic level from *M. septicum* DSM 44393^T^ to be considered to represent a novel species.

**Fig. 3.**
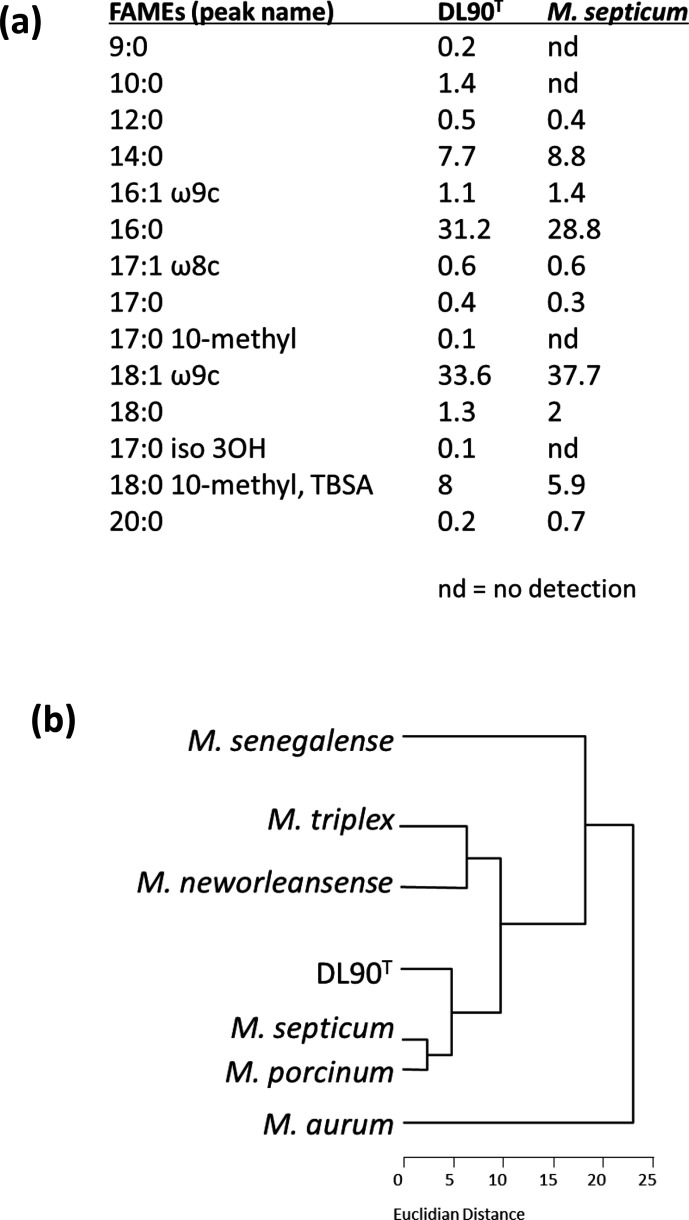
Fatty acid methyl ester (FAME) profiles for DL90^T^ and *Mycolicibacterium septicum* (**a**) and a dendrogram of FAME profiles of similar mycobacterial species grouping closest to DL90^T^ (**b**).

Both DL90^T^ and *M. septicum* colonies have similar smooth circumferences on 7H10 agar supplemented with OADC (Fig. S1), but there are numerous differences between the two strains. When grown on tryptic soy agar, DL90^T^ is much more scalloped compared with *M. septicum* (Fig. S4). While *M. septicum* is fully susceptible to kanamycin (20 μg ml^−1^) on TSA, DL90^T^ is completely resistant. When standing liquid cultures were compared, *M. septicum* biofilm was much thicker and adhered more strongly to glass surfaces than DL90^T^ (Fig. S2). Permissible growth temperature ranges were similar for DL90^T^ and *M. septicum*, however, DL90^T^ grows much faster growth than *M. septicum* in aerated liquid cultures (Fig. S3). Both *M. septicum* and DL90^T^ showed resistance to heat killing in a water bath although DL90^T^ was much more resistant to heat killing at the two temperatures tested (Fig. S5). Arylsufatase activity was detected at 3 days for DL90^T^ while *M. septicum* DSM 44393^T^ was negative. Lastly, the fatty acid methyl esters (FAMEs) profile of DL90^T^ is distinct from that of *M. septicum* (Fig. 3) (Fatty acid tests were performed for DL90^T^ and *M. septicum* at the same time under identical growth conditions.) Specifically, DL90^T^ contains four fatty acids (9 : 0, 10 : 0, 17 : 0 10-methyl, and 17 : 0 iso 3OH) that are not detected in *M. septicum*. Overall, these genetic, biochemical, morphological and physiological features of isolate DL90^T^ distinguish it as representing a novel species that is given then name *Mycolicibacterium nivoides*.

## Description of *Mycolicibacterium nivoides* sp. nov.

*Mycolicibacterium nivoides* (ni.vo′i.des. L. fem. n. *nix* snow; N.L. suff. -*oides* (from Gr. suff. -*eides*, from Gr. n. *eidos*, that which is seen, form, shape, figure), resembling, similar; N.L. neut. adj. *nivoides* snow-like).

Cells are small, Gram-reaction-variable, acid-fast-positive, non-spore-forming, non-motile straight rods. Colonies are dry, rough, flat, non-chromogenic with undulated/scalloped edges on tryptic soy agar. Colonies bind tightly to agar surfaces, and the isolate forms a flocculent, white biofilm on the surface of liquid media with some attachment to glass surfaces. Colonies appear within 3 days of growth on tryptic soy broth with 1.5 % (w/v) agar, with relatively slower growth on 7H10 agar enriched with OADC. Growth is observed at 25–37 °C with optimal growth at 28 °C. No growth is observed at 42 °C although cultures can survive up to a week at 42 °C on agar before resuming growth at lower temperatures. The isolate cannot survive on agar at 45 °C for a week. Modest growth with 5 % (w/v) NaCl. Positive reaction in enzymatic tests for 68 °C catalase, semi-quantitative catalase, urease, 3- and 14-day arylsulfatase activity and 10-day tellurite reduction. Negative reactions for Tween 80 hydrolysis, nitrate reduction, and 3-day tellurite reduction. The 16S rRNA gene is most similar to that of *M. septicum* DSM 44393^T^, but ANI values indicate that this is a novel species. The fatty acid methyl ester (FAME) profile is distinct from that of *M. septicum*.

The type strain is DL90^T^ (=JCM 32796^T^=NCCB 100660^T^) and was isolated from a sphagnum peat bog in northern Minnesota, USA. GenBank accession numbers for the 16S rRNA and the genome of the type strain are MH290160 and CP034072.1, respectively.

## Supplementary Data

Supplementary material 1

## References

[1] Gupta RS, Lo B, Son J (2018). Phylogenomics and comparative genomic studies robustly support division of the genus *Mycobacterium* into an embeded genus *Mycobacterium* and four novel genera. Front Microbiol.

[2] Kazda J (2000). The Ecology of Mycobacteria.

[3] Hannigan GD, Krivogorsky B, Fordice D, Welch JB, Dahl JL (2013). *Mycobacterium minnesotense* sp. nov., a photochromogenic bacterium isolated from sphagnum peat bogs. Int J Syst Evol Microbiol.

[4] Telenti A, Marchesi F, Balz M, Bally F, Böttger EC (1993). Rapid identification of *Mycobacteria* to the species level by polymerase chain reaction and restriction enzyme analysis. J Clin Microbiol.

[5] Tran PM, Dahl JL (2016). *Mycobacterium sarraceniae* sp. nov. and *Mycobacterium helvum* sp. nov., isolated from the pitcher plant *Sarracenia purpurea*. Int J Syst Evol Microbiol.

[6] Bolger AM, Lohse M, Usadel B (2014). Trimmomatic: a flexible trimmer for Illumina sequence data. Bioinformatics.

[7] Wick RR, Judd LM, Gorrie CL, Holt KE (2017). Unicycler: resolving bacterial genome assemblies from short and long sequencing reads. PLoS Comput Biol.

[8] Bankevich A, Nurk S, Antipov D, Gurevich AA, Dvorkin M (2012). SPAdes: a new genome assembly algorithm and its applications to single-cell sequencing. J Comput Biol.

[9] Kumar S, Stecher G, Tamura K (2016). mega7: molecular evolutionary genetics analysis version 7.0 for bigger datasets. Mol Biol Evol.

[10] Altschul SF, Gish W, Miller W, Myers EW, Lipman DJ (1990). Basic local alignment search tool. J Mol Biol.

[11] Lee MD (2019). GToTree: a user-friendly workflow for phylogenomics. Bioinformatics.

[12] Hyatt D, LoCascio PF, Hauser LJ, Uberbacher EC (2012). Gene and translation initiation site prediction in metagenomic sequences. Bioinformatics.

[13] Eddy SR (2011). Accelerated profile HMM searches. PLoS Comput Biol.

[14] Edgar RC (2004). Muscle: a multiple sequence alignment method with reduced time and space complexity. BMC Bioinformatics.

[15] Capella-Gutierrez S, Silla-Martinez JM, Gabaldon T (2009). TrimAl: a tool for automatic alignment trimming. Bioinformatics.

[16] Nguyen L-T, Schmidt HA, von Haeseler A, Minh BQ (2015). IQ-TREE: a fast and effective stochastic algorithm for estimating maximum-likelihood phylogenies. Mol Biol Evol.

[17] Hug LA, Baker BJ, Anantharaman K, Brown CT, Probst AJ (2016). A new view of the tree of life. Nat Microbiol.

[18] Konstantinidis KT, Tiedje JM (2005). Genomic insights that advance the species definition for prokaryotes. Proc Natl Acad Sci U S A.

[19] Auch AF, von Jan M, Klenk H-P, Göker M (2010). Digital DNA–DNA hybridization for microbial species delineation by means of genome-to-genome sequence comparison. Stand Genomic Sci.

[20] Parks DH, Chuvochina M, Waite DW, Rinke C, Skarshewski A (2018). A standardized bacterial taxonomy based on genome phylogeny substantially revises the tree of life. Nat Biotechnol.

