# Supplementary material for: Mycolicibacterium nivoides sp. nov isolated from a peat bog
Source: Int J Syst Evol Microbiol. 2021 Mar 1;71(3):004438. doi: 10.1099/ijsem.0.004438 (PMC8375421; doi:10.1099/ijsem.0.004438)
Supplement: Supplementary material 1 [file ijsem-71-4438-s001.pdf]

Supplemental Materials

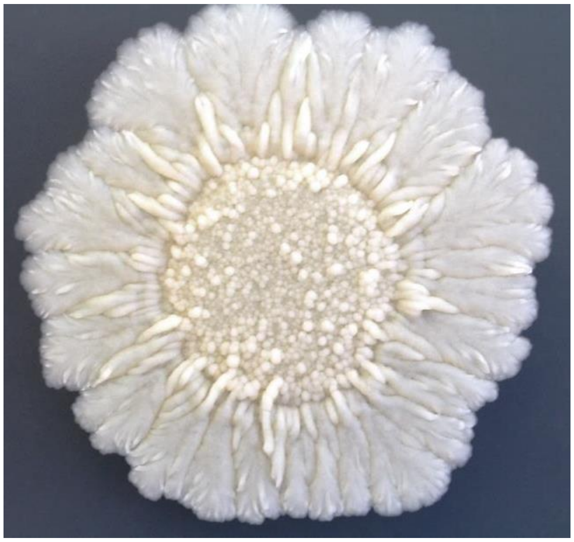

Fig.S1. Appearance of *M. nivoides* isolate DL90^T colony growing on tryptic soy agar at 32°C for 21 days.

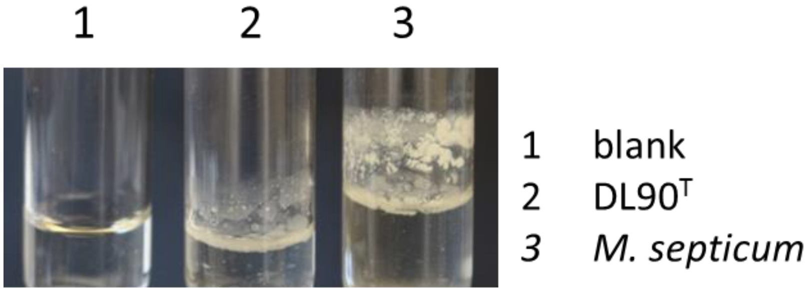

Fig. S2. Biofilm formation of bacteria growing in 7H9 + ADC without Tween 80. Cultures were grown in glass test tubes at room temperature for 7 days.

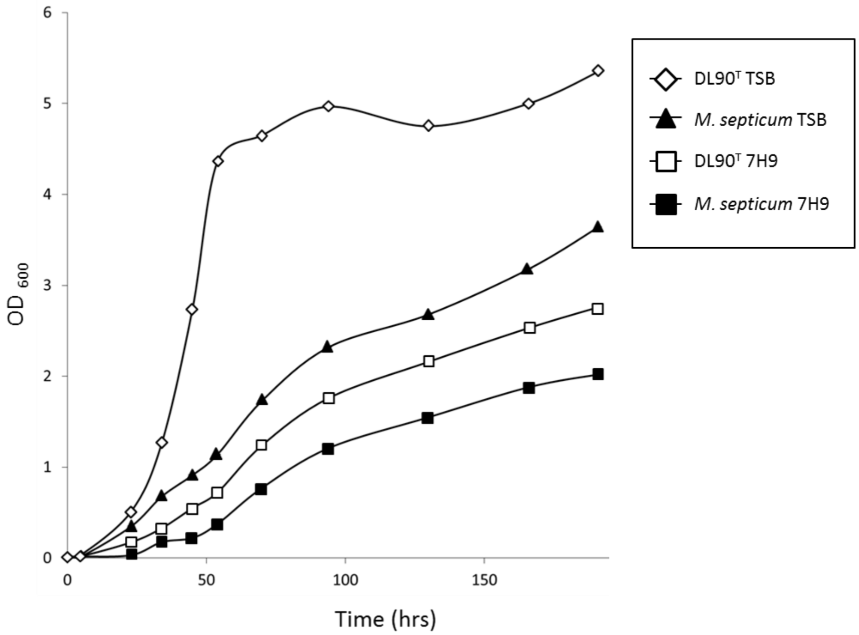

Fig. S3. Growth curves for DL90^{T} and *M. septicum* in either 7H9 + ADC + Tween 80 (7H9) or tryptic soy broth + Tween 80 (TSB) at 32°C in a shaking incubator. Culture densities (optical density OD_{600}) was measured over time.

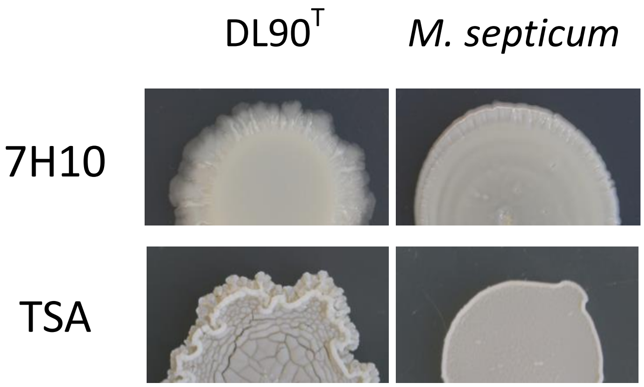

Fig. S4. Comparison of mycolicibacterial species growing on 7H10 + OADC or on tryptic soy agar.

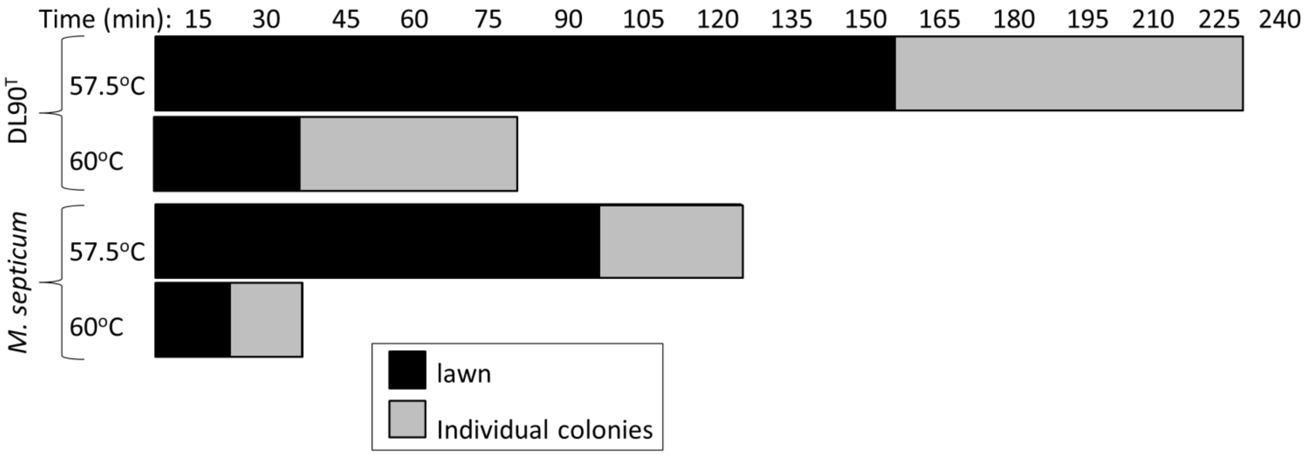

Fig. S5 Survival of mycolicibacterial species in water bath with increasing exposures to heat before spotting onto nutrient agar (NA) plates and monitoring for growth. Stains were grown on solid nutrient agar for one week before re suspending in dH_2O with Tween 80 (0.05% w/v) and equilibrating at cell densities of OD_{600} 4.0. Aliquots of cells (400 µl) were incubated at either 57°C or 60°C with 20-µl aliquots removed and spotted on NA plates every 15 min.

Table S1 GenBank accession numbers used in phylogenetic studies.

**Strain**	**16S rRNA gene**
Mycolicibacter arupense GTC 2730[T]	AB239926.1
Mycobacterium asiaticum ATCC 25276[T]	NR_041901.1
Mycolicibacterium aurum ATCC 23366[T]	NR_116555.1
Mycobacterium chelonae ATCC 35752[T]	NR_119149.1
Mycolicibacterium chitae CIP 105383[T]	AF547910.1
Mycolicibacterium crocinum JCM 16369[T]	MH169218.1
Mycolicibacterium diernhoferi ATCC 19340[T]	NR_041903.1
Mycolicibacterium fortuitum subsp. *fortuitum* DSM 46621[T]	NR_114893.1
Mycolicibacterium gilvum ATCC 43909[T]	NR_118915.1
Mycobacterium gordonae ATCC 14470[T]	NR_118331.1
Mycolicibacter hiberniae ATCC 9874[T]	NR_026092.1
Mycobacterium intermedium CIP 104542[T]	AF547938.1
Mycobacterium kansasii CCUG 32245[T]	FR822390.1
Mycobacterium lentiflavum ATCC 51985[T]	NR_041898.1
Mycolicibacterium mucogenicum ATCC 49650[T]	NR_042919.1
Mycolicibacterium neworleansense ATCC 49404[T]	NR_042914.1
Mycobacterium palustre DSM 44572[T]	AY943210.1
Mycolicibacterium peregrinum ATCC 14467[T]	NR_114447.1
Mycolicibacterium porcinum ATCC 33776[T]	NR_114657.1
Mycobacterium scrofulaceum ATCC 19981[T]	NR_117218.1
Mycolicibacterium senegalense NCTC 10956[T]	LT558839.1
Mycolicibacterium septicum DSM 44393[T]	NR_042916.1
Mycobacterium simiae ATCC 25275[T]	AJ439538.2

Mycobacterium triplex ATCC 700071^T	NR_117226.1
Mycolicibacillus triviale ATCC 23292^T	NR_119192.1
Mycolicibacterium nivoides DL90^T JCM 32796^T and NCCB 100660^T	MH290160
Corynebacterium diptheriae gravis NCTC 13129^T	GQ118344

Table S2. Comparison between genomes of DL90^T and other characterized species that group together based upon 16S rRNA gene relatedness.

**Reference genome**	**No. of genes**	**Comparison genome**	**Genbank Acc. No.**	**No. of genes**	**Mean AAI**	**Std AAI**	**No. orthologous genes**	**Orthologous fraction (OF)**	**Digital DNA hybridization (%)**
DL90^T	6680	*M. septicum*	CBMO010000001	6601	95.93	6.83	5234	80.65	60.9
-	-	*M. boenickei*	FUWC01000001	6266	91.13	8.78	5144	82.09	36.3
-	-	*M. neworleansense*	CWKH01000001	6007	90.91	8.55	5125	85.32	35.6
-	-	*M. porcinum*	OLMG01000001	6656	90.86	8.68	5433	81.63	35.6
-	-	*M. vulneris*	CCBG010000001	6662	90.84	8.75	5430	81.51	35.7
		M. setense	JTJW01000001	5957	90.04	8.91	4944	82.99	33.8
-	-	*M.* sp. (ex Dasyatis americana)	MAIH01000001	5634	89.95	9.15	4185	74.28	33.6
-	-	*M. farcinogenes*	CCAY010000001	5808	89.76	9.69	4695	80.84	32.8
-	-	*M. peregrinum*	LN879423	6899	89.58	9.61	5174	77.46	33.3
-	-	*M. senegalense*	LDPU01000001	6691	89.57	10.1	4857	72.71	32.8
-	-	*M. conceptionense*	CTEF01000001	7108	89.4	10.3	4119	61.66	32.8
-	-	*M. fortuitum*	CP011269	6005	89.38	9.44	4953	82.48	31.3
-	-	*M. houstonense*	FJVO01000001	6407	88.29	9.88	4529	70.69	31.1
